# Predicting Binding to P-Glycoprotein by Flexible Receptor Docking

**DOI:** 10.1371/journal.pcbi.1002083

**Published:** 2011-06-23

**Authors:** Elena Dolghih, Clifford Bryant, Adam R. Renslo, Matthew P. Jacobson

**Affiliations:** 1 Department of Pharmaceutical Chemistry, University of California, San Francisco, San Francisco, California, United States of America; 2 Small Molecule Discovery Center, University of California, San Francisco, San Francisco, California, United States of America; University of Houston, United States of America

## Abstract

P-glycoprotein (P-gp) is an ATP-dependent transport protein that is selectively expressed at entry points of xenobiotics where, acting as an efflux pump, it prevents their entering sensitive organs. The protein also plays a key role in the absorption and blood-brain barrier penetration of many drugs, while its overexpression in cancer cells has been linked to multidrug resistance in tumors. The recent publication of the mouse P-gp crystal structure revealed a large and hydrophobic binding cavity with no clearly defined sub-sites that supports an “induced-fit” ligand binding model. We employed flexible receptor docking to develop a new prediction algorithm for P-gp binding specificity. We tested the ability of this method to differentiate between binders and nonbinders of P-gp using consistently measured experimental data from P-gp efflux and calcein-inhibition assays. We also subjected the model to a blind test on a series of peptidic cysteine protease inhibitors, confirming the ability to predict compounds more likely to be P-gp substrates. Finally, we used the method to predict cellular metabolites that may be P-gp substrates. Overall, our results suggest that many P-gp substrates bind deeper in the cavity than the cyclic peptide in the crystal structure and that specificity in P-gp is better understood in terms of physicochemical properties of the ligands (and the binding site), rather than being defined by specific sub-sites.

## Author Summary

With many drugs failing in the preclinical stages of drug discovery due to undesirable ADMETox (absorption, distribution, metabolism, excretion and toxicity) properties, improvement of these properties early on in the process, alongside the optimization of the compound activity, is emerging as a new focus in the pharmaceutical field. One of the key players affecting pharmacokinetic profiles of many clinically relevant compounds is an active efflux transporter, P-glycoprotein. Expressed predominantly at various physiological barriers, it can influence drug absorption (intestinal epithelium, colon), drug elimination (kidney proximal tubules) and drug penetration of the blood-brain barrier (endothelial brain cells). Moreover, its increased expression in cancer cells has been linked to resistance to multiple drugs in tumors. In this study we describe a computational approach that allows prediction of which compounds are more likely to interact with P-gp. We have tested the ability of this method to differentiate between binders and nonbinders of P-gp by using consistently measured *in vitro* experimental data. We also implemented a blind test on a series of peptidic cysteine protease inhibitors with encouraging outcome. Overall, our results suggest that this method provides a qualitative, quick, and inexpensive way of evaluating potential drug efflux problem at the early stages of drug development.

## Introduction

P-glycoprotein (P-gp) is an ATP-dependent transport protein that is selectively expressed at entry points of xenobiotics in tissues such as the intestinal epithelium, capillary brain endothelium, and kidney proximal tubules among others [1]. Acting as an efflux pump, it prevents exogenous substances from entering sensitive organs and, as such, plays a key role in the absorption and blood-brain barrier penetration of many drugs, affecting their distribution and elimination [2], [3]. Moreover, overexpression of this protein, also known as MDR1, has been linked to multidrug resistance (MDR) in cancer tumor cells where higher levels of the protein result in increased efflux of chemotherapeutic compounds [4]. Finally, there is also accumulating evidence that P-gp, in addition to its role in drug transport, may transport endogenous molecules such as signaling lipids, and play a role in tumor biology and cancer progression [5].

A major hurdle in the drug discovery process [6], [7], P-gp has inspired the development of several assays aimed at identifying its substrates [8], [9]. One widely used assay, the monolayer efflux ratio (ER) assay, measures transport rates of molecules in different directions across a single layer of specialized cells. The ratio or difference of the two rates, basal-to-apical and apical-to-basal, is used to identify P-gp substrates. Another commonly used assay, aimed at identifying P-gp inhibitors as well as substrates, is the calcein-AM (CAM) inhibition assay, in which accumulation of the fluorescent calcein molecule inside the cells indicates an interaction between P-gp and the molecule being tested.

Despite being widely used, both assays have limitations [10]. For example, the monolayer efflux assay may fail to identify P-gp substrates with high passive permeability (>300 nm/s) because efflux by P-gp can be masked by the high diffusion rate of the compounds through the membrane. There is also no standard value of the efflux ratio used to distinguish substrates from nonsubstrates, with cutoff values from 1.5 to 3 being used [11], [12], [13], [14]. Because the CAM assay is based on the competitive inhibition of calcein transport by compounds that interact with P-gp, the assay may not detect P-gp substrates with low passive membrane diffusion rates that reach the P-gp binding site at a much slower rate than the fluorescent compound. Both assays are also expensive and time consuming, and results in different labs can vary significantly. For example, midazolam has been identified as a nonsubstrate [13], an inhibitor [8], [11], a substrate [15] and an inducer [16] in different studies. Doxorubicin, resistance to which has been linked to P-gp overexpression, is another example [17]. The drug has been cited repeatedly as a classical P-gp substrate [18], [19]; however, it has also been classified as a nonsubstrate by several *in vitro* studies [8], [13].

To complement experimental assays, several *in silico* methods have been developed to predict P-gp binding. Among them, pharmacophore models based on anywhere from two [20] to nine [10] features to an ensemble of 100 pharmacophores [21] have been generated. Other approaches have included quantitative structure-activity relationship (QSAR) models and machine-learning algorithms, some of them incorporating up to 70 descriptors [22], [23], [24]. Even though several of the methods report sensitivity of 80% or higher, the extraordinary chemical diversity of the P-gp substrates, reflected by the large numbers of pharmacophores and descriptors, have frustrated efforts to make sense of the chemical data. A multitude of theories about the number, sizes, and locations of the binding sites has further complicated the issue [25], [26], [27], [28].

The recent publication of the mouse P-gp crystal structure [29] (87% identical amino acid sequence to human P-gp) presents an opportunity to develop new prediction methods that take advantage of the receptor structural information to not only identify molecules that bind to P-gp but also to guide chemical optimization, e.g., to attempt to modify interactions with the protein. Among the three published structures, two were crystallized with stereoisomers of a cyclic inhibitor, QZ59, that defines the drug-binding cavity. All structures are in an inward-facing conformation that is believed to be relevant for initial substrate recognition.

Located in the transmembrane region, the large and hydrophobic drug-binding pocket is lined with various aromatic side chains and has no clearly defined sub-sites. Taking into account the well-known ability of the protein to accommodate ligands of various shapes and sizes, such an arrangement strongly supports the “induced fit” ligand binding model proposed by Loo *et al.* [30]. This, combined with the relatively low resolution of the structure, suggests that it is essential to treat the binding site as flexible while modeling binding site interactions, which we demonstrate explicitly in our results here. In this study, we employ a flexible receptor docking method [31], together with scoring methods that include the Glide XP scoring function [32] and a molecular mechanics scoring function with generalized Born implicit solvent (MM-GB/SA) [33], [34], to develop a new prediction algorithm for P-gp binding specificity. We benchmark the method in several ways, including a blind test on a series of peptidic cysteine protease inhibitors, confirming the ability to predict compounds more likely to be P-gp substrates. We also apply this approach to evaluate the ability of P-gp to discriminate endogenous vs. exogenous compounds, and to predict that several endogenous metabolites may be P-gp substrates. Overall, our results suggest that specificity in P-gp is better understood in terms of physicochemical properties of the ligands (and the binding site), rather than being defined by specific sub-sites. We also suggest that many P-gp substrates bind deeper in the cavity than the cyclic peptide in the crystal structure.

## Methods

### Computational Methods

All molecular docking calculations were performed using the mouse P-glycoprotein crystal structure (Protein Data bank [PDB] code 3G60). BLAST [35] sequence alignment with human P-gp revealed 87% overall sequence identity and ∼100% identity within the binding cavity with the exception of mSer725/hAla729 directly facing the binding cavity. The docking calculations were performed using Glide (version 5.6) [36] with the OPLS2005 force field [37], [38]. The receptor structure was prepared and minimized within the Protein Preparation Wizard.

For rigid docking, a rigid receptor grid defined by a 10×10×10 Å inner box was generated. The docking site was either defined by the centroid of the co-crystallized ligand, QZ59-RRR, with a center at (19.1, 52.3, −0.3) Å or defined higher than the original ligand with the center at (19.0, 46.0, −6.0) Å (same as in induced fit docking, see below). All ligands were docked in both standard precision (SP) Glide and extra precision (XP) Glide modes (Figure S1).

Flexible receptor docking was performed using a multi-stage induced fit docking protocol (IFD) [31] as implemented in Schrödinger Suite 2010. Briefly, in the first stage, the van der Waals radii of protein and ligand are scaled by a factor of 0.5 and ligands are docked into the receptor using the default Glide SP mode. Next, Prime is used to predict and optimize selected protein side chains (details below). Finally, the poses are scored and filtered, after which ligands are redocked using Glide XP mode and scored. Final scoring in this work was implemented using the extra precision (XP) Glide [32] scoring function and an MM-GB/SA [33], [34]rescoring function.

The specific protocol was developed and refined using well-known P-gp substrates and inhibitors from Table S1. Since no binding modes are known for any compounds (with the exception of QZ59), optimal parameters were selected based on binding scores and the ability to distinguish binders from non-binders (as described below). The parameters we varied included the inner box coordinates, van der Waals radii scaling, the number of poses saved, and the number and identity of ‘trimmed’ (mutated temporarily to Ala) residues in the initial docking stage. Specifically, we chose to delete the side chains of Phe71, Phe332, and Phe728 in the 1^st^ docking stage. These three residues are located in the center of the cavity, and were most responsible for preventing potent inhibitors from achieving good scores, by binding deeper in the cavity.

In the primary IFD round, a 10×10×10 Å inner box with coordinates (19.0, 47.0, −6.0) Å was used, which is centered deeper in the binding cavity than the cyclic peptide in the crystal structure, and roughly centered on the docked poses of the initial test set. At this stage, all residues lining the cavity were optimized by Prime [39](Table S2), whereas in the following IFD round, only residues within 5 Å of each ligand were minimized. The number of poses saved during the initial docking was set to 100. For all subsequent docking calculations inner box coordinates (19.0, 46.0, −6.0) Å were used. For each ligand, up to 20 top poses were saved and scored with the Glide XP function and MM-GB/SA.

Ligand coordinates were obtained from the DrugBank [40] and PubChem Compound (http://pubchem.ncbi.nlm.nih.gov/) databases and processed using the *Ligprep* 2.4 module. The parameters were assigned based on the OPLS2005 force field. For the QZ59-RRR ligand, selenium atoms were replaced with sulfur atoms. For molecules with stereocenters, only the known active forms were docked. For drugs used as racemic mixtures, both stereoisomers were investigated. The isomer with the more favorable docking score was used in the data analysis. Ionization states were assigned by *Epik*, and groups with pKa between 5 and 9 were treated as neutral while those outside the range were treated as charged.

Initial testing of our approach was conducted with two datasets. One of them was comprised of 24 well-known P-gp binders from Table 1 of the review article by Hennessy *et al.* [41] and 102 endogenous molecules selected from the KEGG database [42] to represent different classes of biological compounds. The rationale for this first test was that most endogenous molecules would not be effluxed by P-gp, providing insight into how P-gp discriminates between endogenous and exogenous molecules. The second dataset was based on the Doan *et al.* study of FDA approved drugs [11] that generated consistent experimental data from the monolayer efflux ratio as well as the calcein-inhibition assays. We used the intersection of the results in the two assays to define sets of compounds that were clear P-gp substrates (i.e., positive in both assays) or non-substrates. We did not consider the compounds that were positive in only one of the two assays. The complete list of compounds and their scores are provided in Tables S1 and Tables S3, S4, and S5.

**Table 1:** Peptidic cysteine protease inhibitors.

Compound	Mean P_app_ (10^−6^ cm/s)		Mean Efflux Ratio	Glide XP (kcal/mol)
	A to B	B to A		
1	24.4	29.6	1.2	−10.9
2	10.6	16.7	1.6	−9.9
3	21.8	30.9	1.4	−12.7
4	19.6	31.8	1.6	−12.9
5	20.3	36.4	1.8	−13.0
6	4.75	42.8	9.0	−14.6
7	10.3	45.5	4.4	−13.2
8	14.5	34.2	2.4	−14.2
9	0.51	2.9	5.8	−12.3
10	8.41	25.8	3.1	−11.0

### Synthetic Methods

Reagents and solvents were purchased from Aldrich Chemical, Alfa Aesar, Chem Impex international or TCI America and used as received. Reactions were carried out under an argon atmosphere in oven-dried glassware using anhydrous solvents from commercial suppliers. Air and/or moisture sensitive reagents were transferred via syringe or cannula and were introduced into reaction vessels through rubber septa. Solvent removal was accomplished with a rotary evaporator at ∼10–50 Torr. Automated column chromatography was carried out using a Biotage SP1 system and silica gel cartridges from Biotage or Silicycle. Analytical TLC plates from EM Science (Silica Gel 60 F_254_) were employed for TLC analyses. ^1^H NMR spectra were recorded on a Varian INOVA-400 400 MHz spectrometer.

Analogs **1** [43], [44], **3**, **6**, and **9** were synthesized in one step from commercially available *N*-benzyloxycarbonyl (Cbz) protected amino acids according to the following general procedure. A solution of the *N*-benzyloxycarbonyl protected L-amino acid (0.33 mmol) in 2 mL of DMF was treated with aminoacetonitrile bisulfate (0.37 mmol, 1.1 equiv.), 1-hydroxybenzotriazole (0.33 mmol, 1.0 equiv), *N*-(3-dimethylaminopropyl)-*N′*-ethylcarbodiimide hydrochloride (0.67 mmol, 2.0 equiv.), and *N,N*-diisopropylethylamine (2.0 mmol, 6.0 equiv.). The reaction was stirred at room temperature and monitored until judged complete by TLC or HPLC. The reaction mixture was then poured into ethyl acetate and the resulting organic solution washed in succession with aqueous 1 N HCl (for non-basic analogs only), 50% aqueous NaHCO_3_, saturated aqueous NaCl, and then dried (MgSO_4_), filtered, and concentrated. The crude product thus obtained was purified using automated silica gel flash chromatography (Biotage SP1, ethyl acetate-hexane) to afford the desired products.

Analogs **2**, **4**, **5**, **7**, and **10** were synthesized in two steps from N-(benzyloxycarbonyl)-L-serine lactone [45] according to the following procedure. A solution of *N*-(benzyloxycarbonyl)-L-serine lactone (0.45 mmol) in 2 mL of acetonitrile was added dropwise to a solution of the relevant amine or *N*-trimethylsilylamine (1–10 equivalents depending on the amine, see below) in ∼3 mL of acetonitrile. The reaction was monitored at room temperature or in some cases heated at 50°C, depending on the reactivity of the amine (see below). When the reaction was judged complete by TLC or HPLC, the reaction mixture was concentrated and the desired amino acid separated from undesired amide side product in one of the following ways. For the amino acid leading to **2**, the crude product was partitioned between ethyl acetate and water and the water phase (containing the desired product) was then lyophilized. For the amino acids leading to **4** and **5**, purification by automated silica gel flash chromatography (Biotage SP1, methanol-dichloromethane) afforded the desired amino acids. For intermediate amino acids leading to **7** and **10**, the crude residue was partitioned between dichloromethane and 1 N aqueous NaOH, followed after phase separation by acidification of the aqueous phase with 1 N HCl to effect precipitation of the amino acid, which was collected on a filter, washed with cold water, and dried. The procedures described above provided the desired amino acids in sufficient purity for use in the subsequent coupling reaction with aminoacetonitrile, which was carried out according to the general coupling protocol described for analogs **1**, **3**, **6**, and **9** above.

Analog **8** was prepared in three steps by reaction of indoline with *N*-(benzyloxycarbonyl)-L-serine lactone as described above, using automated silica gel flash chromatography (Biotage SP1, methanol-dichloromethane) to isolate the desired amino acid. The amino acid intermediate was coupled to aminoacetonitrile according to the general procedure and finally, the resulting indoline product was oxidized to the desired indole **8** by reaction with 1.05 equivalents of 2,3-dichloro-5,6-dicyano-1,4-benzoquinone (DDQ) in dichloromethane for 30 minutes. The final product was purified by automated silica gel flash chromatography (Biotage SP1, ethyl acetate-hexane).

Additional details and NMR data are provided in Supplementary Methods (Text S1).

### MDCK-MDR1 Monolayer Permeability Assay

Permeability measurements were performed by Wuxi Apptec, using the following procedures.

MDCK-MDR1 cells (obtained from Piet Borst at the Netherlands Cancer Institute) were seeded onto polyethylene membranes (PET) in 96-well BD insert systems at 2×10^5^ cells/cm^2^ for 4–6 days to obtain confluent cell monolayer formation. Test compounds were diluted with the transport buffer (HBSS, pH 7.4) from a 10 mM stock solution to a concentration of 2 µM and applied to the apical (A) or basolateral (B) side of the cell monolayer. Permeation of the test compounds from the A to B direction or B to A direction was determined in triplicate over a 150-minute incubation at 37°C and 5% CO_2_ with a relative humidity of 95%. In addition, the efflux ratio of each compound was also determined. Test and reference compounds were quantified by LC-MS/MS analysis based on the peak area ratio of analyte/IS.

The apparent permeability coefficient P_app_ (cm/s) was calculated using the equation:
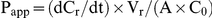
where dC_r_/dt is the cumulative concentration of compound in the receiver chamber as a function of time (µM/s); V_r_ is the solution volume in the receiver chamber (0.075 mL on the apical side, 0.25 mL on the basolateral side); A is the surface area for the transport, i.e. 0.084 cm^2^ for the area of the monolayer; C_0_ is the initial concentration in the donor chamber (µM).

The efflux ratio (ER) was calculated using the equation:
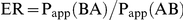



Percent recovery was calculated using the equation:
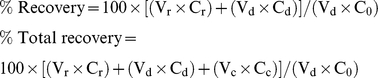
Where V_d_ is the volume in the donor chambers (0.075 mL on the apical side, 0.25 mL on the basolateral side); C_d_ and C_r_ are the final concentrations of transport compound in donor and receiver chambers, respectively. C_c_ is the compound concentration in the cell lysate solution (µM), and V_c_ is the volume of insert well (0.075 mL in this assay).

Permeability determinations were performed in triplicate and are reported as mean values. The mean total recovery was greater than 90% for all compounds tested (compounds **1**–**10**).

## Results

**Figure 1:**
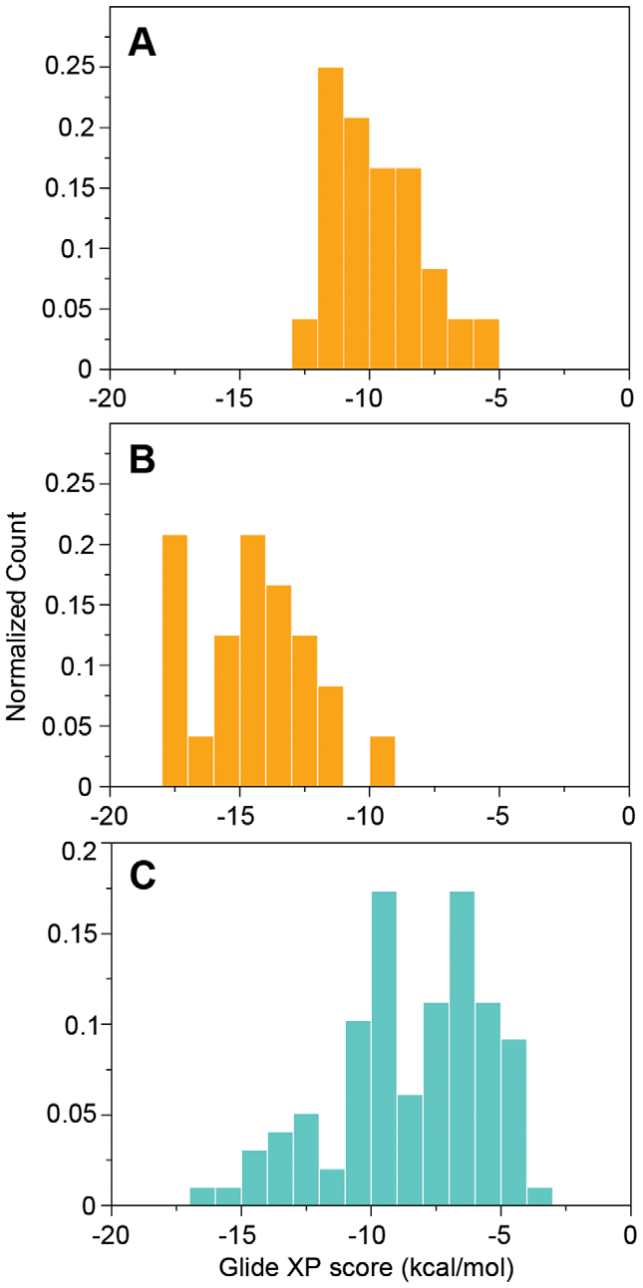
Distributions of docking scores (Glide XP). Metabolites/P-gp binders set: **A**) Rigid docking, binders; **B**) Flexible docking, binders; **C**) Flexible docking, metabolites.

**Figure 2:**
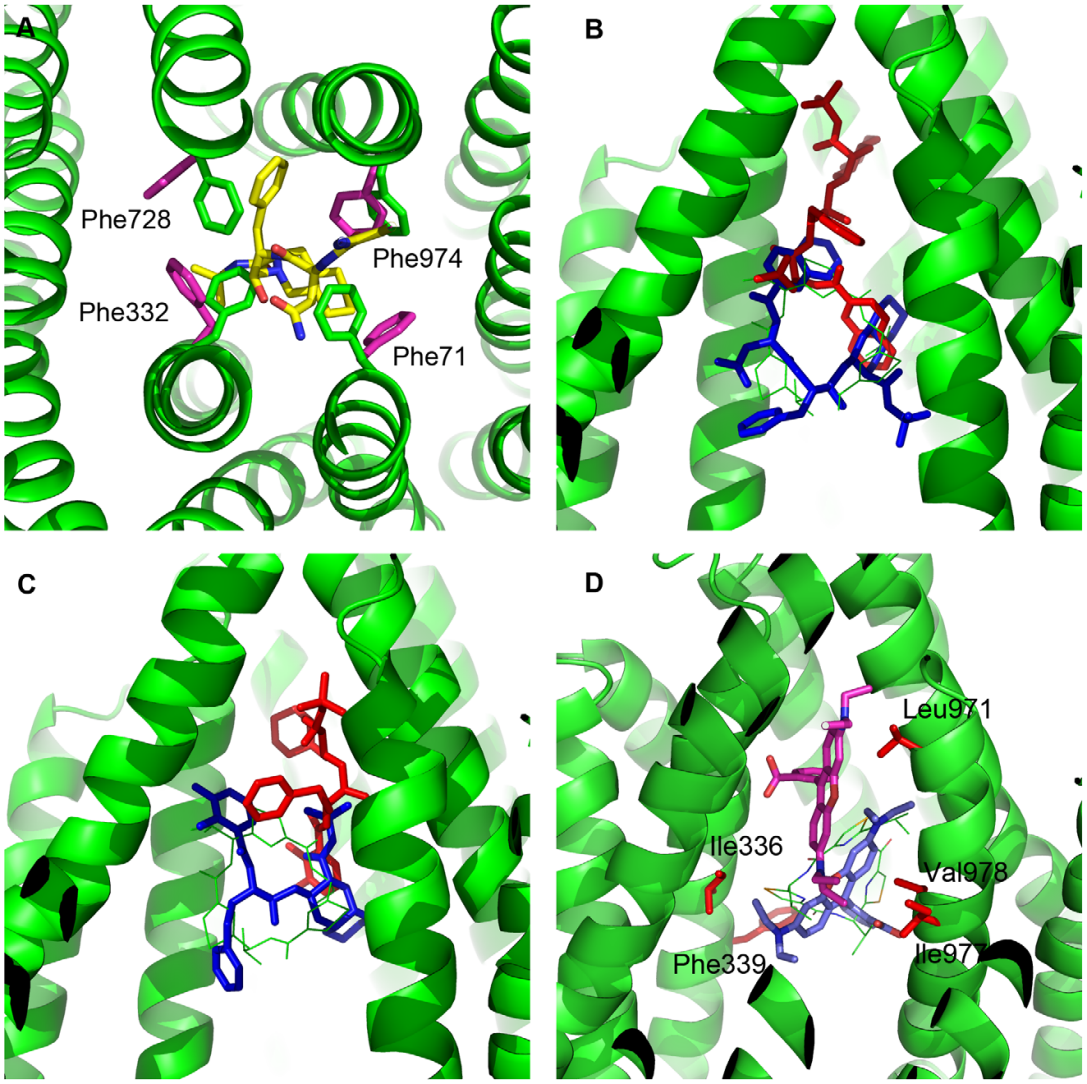
Flexible versus rigid docking. **A**) View of P-gp from above (i.e. viewed from the luminal face, perpendicular to the membrane plane) of the top-scored saquinavir pose (yellow) from the primary round of the flexible receptor docking. Phe71, Phe332, and Phe728 were mutated to Ala in the 1^st^ Glide docking stage. The final positions of the three residues and Phe974 are shown in pink, while those of the original crystal structure are in green. **B**) Top-scored poses from second round of flexible receptor docking (red), Glide XP = −17.8 kcal/mol compared to top the pose from rigid docking (blue), Glide XP = −10.0 kcal/mol for saquinavir. **C**) Analogous results for nelfinavir. Flexible docking (red), Glide XP = −15.6 kcal/mol; rigid docking (blue), Glide XP = −8.9 kcal/mol. **D**) Rhodamine B. Flexible receptor docking (pink), Glide XP = −15.3 kcal/mol. Rigid docking (blue), Glide XP = −5.4 kcal/mol. In red, are residues shown experimentally to interact with a cysteine analogue of rhodamine B. QZ59-RRR is shown for reference in light green.

We initially developed the docking strategy using a number of well-known P-gp substrates and inhibitors. Specifically, we used a set of 24 known binders (Hennessy *et al.* [41], Table 1) that included, among others, HIV protease inhibitors, anthracyclines, vinca alkaloids, and taxanes (Table S1). Initial docking using a rigid receptor and docking box coordinates centered on the co-crystallized ligand generated poses that largely overlapped with the coordinates of the cyclic peptide in the crystal structure, with most of the compounds showing extensive exposure to solvent at the base of the cavity. By contrast, the flexible-receptor docking protocol resulted in ligands receiving much more favorable docking scores (Figure 1) as well as ligand poses in which the ligands bound much ‘deeper’ in the cavity (Figure 2), with little solvent exposure.

Only side chains in the binding site were treated as flexible (Table S2), and the conformational changes that allowed the ligands to bind more deeply in the cavity were modest. The most important conformational changes were those of the side chains of Phe71, Phe332, and Phe728, which are located in the center of the cavity. As shown in Figure 2A, the rotamer changes in these side chains result in a more open cavity than that in the initial crystal structure. Representative top poses and Glide XP binding scores of some of the compounds from the final IFD round are shown in Figures 2B and 2C. Additional scores are provided in , and are represented as a histogram in Figure 1, highlighting the much more favorable docking scores achieved with flexible-receptor docking. Below, we also demonstrate that the flexible receptor approach greatly improves the ability to discriminate binders from non-binders.

In all of this work, we used two different scoring functions to rank compounds, Glide XP and a molecular mechanics based scoring function (MM-GB/SA), in addition to the default Glide SP scoring function. The results using Glide XP and MM-GB/SA scoring were, on average, remarkably similar, considering the very different functional form and methods of parameterization. Both scoring functions performed much better than Glide SP in distinguishing binders from non-binders. (On the other hand, the Glide XP and MM-GB/SA scoring functions did not always identify the same poses as top-ranked; see, e.g., doxorubicin in Figure S2. Given the size and flexibility of the binding cavity, it is likely that for some, if not all molecules, several binding modes are possible.) For simplicity, we present mainly the results using Glide XP here, and present the remaining results using MM-GB/SA in supplementary tables and figures, in part because the results with Glide XP are slightly better in some cases. This is not altogether surprising because the molecular mechanics scoring function has been useful primarily in ranking compounds that are chemically similar, e.g., congeneric series, and the series of compounds we use in most of the tests here are quite diverse. However, the similarity of the results using the very different scoring functions is striking, and we use the results with MM-GB/SA scoring to investigate, for example, the role of desolvation in binding by P-gp.

Also shown in Figure 2D are the rigid- and flexible-receptor poses of a well-known (non-drug) P-gp substrate, rhodamine B, the binding mode of which has been partially elucidated by experimental data obtained using its Cys-linked analog. The flexible-receptor pose selected by Glide XP is qualitatively consistent with the experimental data in that the molecule is reasonably close to residues facing the binding cavity and shown to interact with the ligand [26], [46]. By comparison, the pose obtained by rigid docking appears less consistent with the experimental data and also has a much less favorable docking score (−5.4 kcal/mol, vs. −15.3 kcal/mol for the flexible receptor pose).

**Figure 3:**
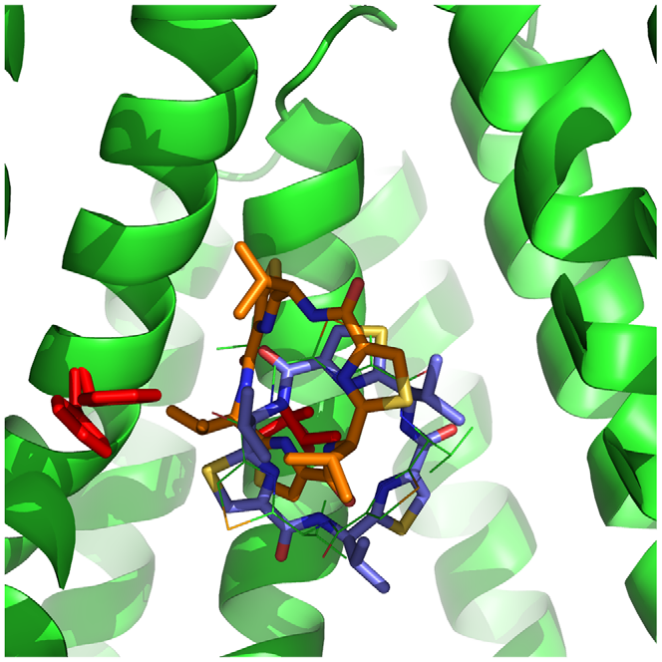
Flexible and rigid docking results for QZ59-RRR. Flexible receptor docking (orange), Glide XP = −10.2 kcal/mol. Rigid docking (blue), Glide XP = −8.4 kcal/mol. QZ59-RRR from the crystal structure is shown for reference in light green. In red are two residues believed to be important for ligand binding: Phe724 and Val978.

As an additional control, we docked QZ59-RRR back into the crystal structure using both rigid and flexible docking protocols. The results are illustrated in Figure 3. Rigid docking reproduced the binding mode of QZ59, but the molecule was ‘flipped’ in comparison to its position in the crystal structure. Induced fit docking produced a similar pose with the molecule slightly shifted upwards from the original position. (The shift was seen regardless of whether the docking box was centered on the original ligand position or shifted deeper into the cavity). The ligand still maintained contact with Phe724 and Val978 deemed to be important for drug binding (as discussed in Aller paper), as well as with the majority of the hydrophobic residues indicated to be within 4–5 Å of the crystal pose (Table S6). The flexible docking score in this case, though not particularly high, is also more favorable than that from rigid docking. Based on the IC_50_ value reported for QZ59-RRR inhibition of verapamil-stimulated ATPase activity (4.8±2.6 µM), the ligand is a rather weak inhibitor, which could partially explain the weak binding score. In addition, as discussed in Methods, it was not possible to dock the compound containing selenium atoms, and these were replaced with sulfur.

We next docked a set of 102 common metabolites, comprising representatives of four major classes of biological molecules including carbohydrates, amino acids, fatty acids, and nucleic acids (Table S3). We reasoned that most of these metabolites would be non-binders based on the notion that their efflux would be inefficient to cell function (as would inhibition of P-gp by metabolites). As shown in Figure 1, most of the metabolites did in fact have much less favorable docking scores than the drugs discussed above. However, a small fraction of the metabolites received docking scores similar to those of the drugs. For example, of the 26 drugs in Table S1 known to interact with P-gp, 23 had Glide XP scores of −12 kcal/mol or better, and 15 had scores <−14 kcal/mol. By contrast, 18 of the 102 metabolites received scores more favorable than −12 kcal/mol, and only 8 had scores <−14 kcal/mol. Among these metabolites with favorable scores, we subsequently identified four (thyroxin, vitamin D3, progesterone, and cholesterol) that have been reported to interact with P-gp [47], [48], [49], [50] and reassigned them to the binders set (which had little affect on the ROC-type curve). We also searched for literature data on approximately 20 randomly selected metabolites with less favorable docking scores and were unable to find any evidence of these being P-gp substrates. We also could not identify any direct evidence for other top-scoring metabolites, such as riboflavin, retinol, and leukotriene C4, interacting with P-gp, but it is possible that some of these metabolites are currently unrecognized substrates (or inhibitors). In fact, P-gp has been suggested to export naturally derived toxins in healthy cells [51] as well as to play a role in transport of cancer-signaling lipids [5]. Investigation of a more extensive set of biologically relevant molecules is currently under way.

**Figure 4:**
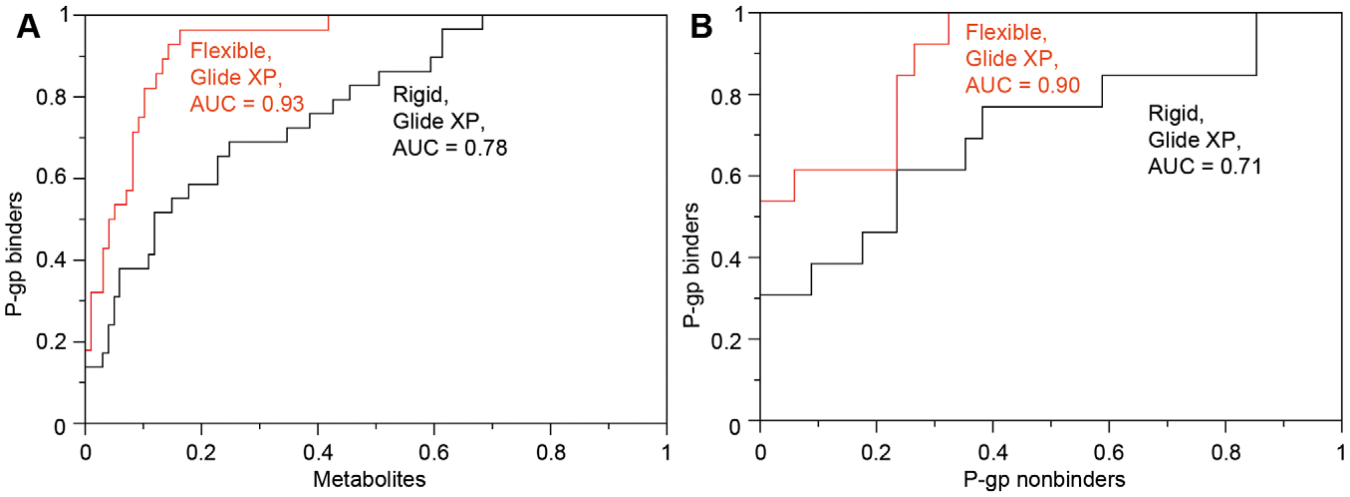
ROC-type curves (Glide XP) for metabolites/P-gp binders set (A) and Doan *et al.* dataset (B).

In the absence of any direct evidence for the other metabolites, we consider them non-binders, and we quantify the ability to discriminate the known binders (26 drugs+4 metabolites) and presumed non-binders (98 metabolites) using an ROC-type curve in Figure 4A. Clearly, the flexible-receptor protocol results in much better discrimination between these two sets of compounds (area under the curve, AUC = 0.93) than the rigid receptor docking, either with the docking box centered on the co-crystallized QZ59 ligand (AUC = 0.78, Figure 4A), or with the docking box shifted deeper into the cavity as in the flexible docking results (AUC = 0.83, Figure S1). The results using flexible-receptor docking and the MM-GB/SA scoring function are very similar in this case, AUC = 0.93 (Figure S3).

Next, we tested the ability to qualitatively reproduce results of *in vitro* assays regularly used to evaluate P-gp binding. For that purpose we selected the Doan *et al.* [11] dataset of FDA approved drugs that included results of the monolayer efflux and CAM inhibition assays. Based on the assay results, we defined P-gp binders (N = 13) as molecules positive for both assays (ER>1.5 and >10% CAM inhibition), and nonbinders (N = 34) as compounds negative for both assays (ER<1.5 and <10% CAM inhibition). The ROC curves obtained with Glide XP and the default treatment of ionization (see Methods) are shown in Figure 4B. The induced fit approach (AUC = 0.90) again outperformed rigid docking (AUC = 0.71), although there is clearly some overlap in the distribution of scores between the binders and non-binders in this set. Some of this overlap is due to the somewhat arbitrary criteria used for distinguishing the sets of compounds, as discussed below.

The results using the MM-GB/SA (AUC = 0.81) scoring function were somewhat worse than using Glide XP (Figure S3), using the default treatment of ionization states. However, when all compounds were docked as neutral species, regardless of their pKa values, the MM-GB/SA scoring function performed much better (AUC = 0.92, Figure S4); the results using the Glide XP scoring function were similar (AUC = 0.92). It is not surprising that the MM-GB/SA scoring function is more sensitive to the treatment of protonation states, since charged compounds have large, unfavorable desolvation energies. It is not completely clear why treating all compounds as neutral results in better discrimination, although we note in this regard that one of the prevalent theories in the field is that P-gp substrates enter the binding cavity from the membrane (where they are assumed to be electrically neutral) rather than directly from the aqueous environment of the cytoplasm [4].

**Figure 5:**
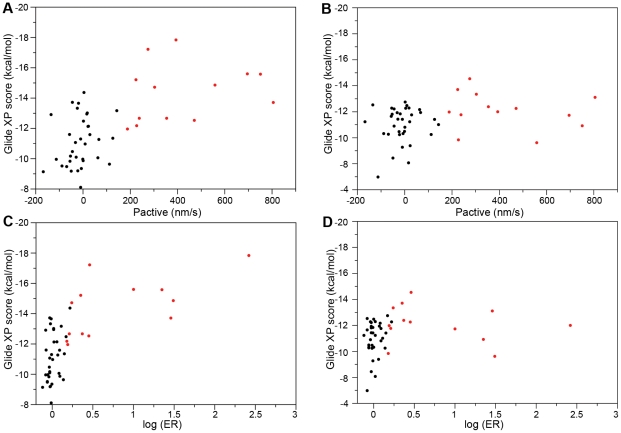
Flexible (A, C) and rigid (B, D) docking binding scores (Glide XP) versus P_active_ and log of efflux ratio. Compounds from Doan *et al.* dataset [11].

It is currently challenging to measure binding affinities to P-gp, and the results of standard assays are generally interpreted qualitatively (i.e., is it a substrate or not), as we have done here. However, the ratio or difference of the rates of permeability in the two directions (basal-to-apical, P_BA_, and apical-to-basal, P_AB_) provides a quantitative measure of how ‘strong’ a substrate a given compound is. Although there is no reason to expect the computed docking scores to necessarily correlate well with these metrics, there is, in fact, a reasonable correlation with both the difference in the rates (P_active_ = P_BA_−P_AB_) and the more commonly used log of the efflux ratio (Figure 5A). (Results using the MM-GB/SA scoring function are qualitatively similar, and again the results using this scoring function are better when all molecules are treated as neutral (Figures S5 and S6). One advantage of representing the data this way is that it avoids somewhat arbitrary criteria used for classifying the compounds as substrates and non-substrates. The plots illustrate the two different, if somewhat overlapping ranges of the binding scores for P-gp binders and nonbinders. When the same data was plotted versus rigid docking scores, the two classes were undistinguishable (Figure 5B). When interpreting these plots, one must keep in mind that efflux ratio values are a result of a complex interplay between the binding affinities of the compounds and kinetic aspects of the P-gp efflux, and may be influenced by rate of passive membrane permeability, and we do not claim that it should be possible to quantitatively predict the efflux ratio based on docking calculations alone.

The P-gp binding site is highly hydrophobic and the ligand desolvation energy may have a significant effect on the ligand binding. To investigate this point, we computed free energy of transferring compounds from water to a low dielectric solvent (chloroform) using an approach described previously for predicting passive membrane permeability [52], [53]. The plot of the P-gp binding scores vs. free energy of desolvation showed no correlation between the two (Figure S7), indicating that binding scores reflect specific interactions with P-gp and are not dominated solely by the polarity/hydrophobicity (quantified using the solvation free energy) of the compounds. Some of these specific interactions are illustrated in Figure S2 and include pi-stacking, cation-pi interactions and hydrogen bonding. These results also suggest that the physicochemical properties of the ligands that define their passive membrane permeability are different from the physicochemical determinants that define their interactions with P-gp. This finding in turn suggests that it might be possible to reduce a ligand's P-gp binding using chemical modifications without dramatically reducing its membrane permeability. We note that the computations we perform here attempt to predict the (path-independent) thermodynamics of transferring a ligand from water to the P-gp binding site, and thus our computations do not provide any direct information about whether the ligand enters the binding site through the membrane, or directly from the cytoplasm.

**Figure 6:**
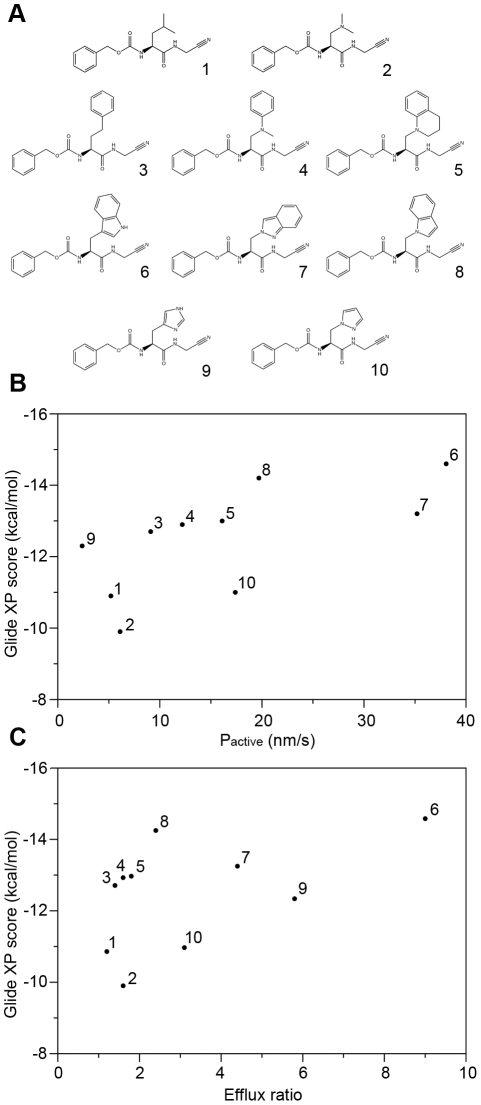
Series of peptidic cysteine protease inhibitors. Compound structures (**A**). Plot of binding scores (Glide XP) vs. P_active_ (**B**) and experimentally measured efflux ratio values (**C**).

Finally, we have performed a first ‘blind’ test of the method, using a series of peptidic cysteine protease inhibitors bearing natural and unnatural amino acid residues. These compounds were originally designed to test hypotheses concerning passive membrane permeability, and those results will be reported elsewhere. However, we also tested the compounds in a cell-monolayer assay (performed by WuXi AppTec), specifically using P-gp transfected MDCK cells. The results are summarized in Table 1 and Figure 6, where we again plot the predicted docking scores for binding to P-gp versus measures of the asymmetry of the permeability across the monolayer. The compound that showed the strongest evidence for P-gp mediated efflux, compound **6**, had an efflux ratio of 9 with moderate passive membrane permeability. Encouragingly, this compound had the most favorable Glide XP score (−14.6) among the series, comparable to many of the P-gp substrates in the benchmarking results discussed above. Similarly, the compound with the least favorable docking score (−9.9, compound **2**) had a much lower efflux ratio (1.6), and the compound with the lowest measured efflux ratio (1.2, compound **1**) had the second lowest docking score (−10.9).

The results are not perfect. Compound **9** has relatively polarized efflux (ER = 5.8), but has a docking score that would classify it only as a ‘possible’ binder (−12.3). However, we note that this compound has 10× lower passive membrane permeability than any other member of the series, P_AB_ = 5×10^−7^ cm/s, making the determination of P-gp mediated efflux more uncertain. In general, the correlation between the docking scores and the experimental results is ‘noisier’ than in the benchmarking study using the Doan *et al.* data set. However, we note that the range of experimental efflux ratios, as well as the range of docking scores, is narrower in this series of peptidic compounds, which is expected due to the compounds being much more chemically similar. As such, it is gratifying to be able to confirm an ability to predict compounds more/less likely to be P-gp substrates, even in the more challenging case of a more chemically homogeneous series, albeit not with ‘quantitative’ accuracy.

In this regard, we again emphasize that, even with perfect ability to predict binding affinities to P-gp (which we certainly do not claim), there is no reason to expect any simple relationship between the docking scores and experimental measures of P-gp mediated efflux. Thus the docking results, as well as the experimental results, should be interpreted in qualitative terms. As general guidelines based on the work presented here, we view a Glide XP docking score of approximately −14 or lower, using the flexible docking protocol, to be a predictor of P-gp interaction, while scores of −12 or greater predict non-interaction. Intermediate scores of approximately −12 to −14 are less conclusive, but many compounds in this range show evidence of being relatively ‘weak’ substrates.

## Discussion

In summary, we have developed an *in silico* method suitable for predicting compounds that are more likely or less likely to interact with P-gp. We tested the ability of this method to differentiate between binders and nonbinders of P-gp using consistently measured experimental data from P-gp efflux and calcein-inhibition assays. Treating the P-gp binding cavity as flexible is critical for obtaining good results. We suspect that this observation reflects intrinsic flexibility of the binding site, but may also be related to the relatively low resolution of the crystal structures; most of the side chains orientations are not well defined by the electron density. Encouragingly, a first ‘blind’ test of the flexible-receptor approach on a series of peptidic protease inhibitors provided additional evidence for the predictive ability of the method.

Overall, our results suggest that specificity in P-gp is better understood in terms of physicochemical properties of the ligands (and the binding site), rather than being defined by specific sub-sites. We also suggest that many P-gp substrates bind deeper in the cavity than the cyclic peptide in the crystal structure. Finally, we also make testable predictions concerning metabolites that may be P-gp substrates.

We have not explored whether we can distinguish substrates and inhibitors of P-gp. Experimentally distinguishing these two classes is not simple, and available evidence suggests that substrates can competitively inhibit efflux of other compounds to varying extents [19]. From the standpoint of the computations, it is clear that both substrates and inhibitors must bind to P-gp, presumably in the ‘inward’ configuration represented in the crystal structures. We speculate that the mode of binding (i.e., where in the binding site the compounds bind) might relate to these complicated considerations. However, the biophysical basis of coupling between ligand binding and ATP hydrolysis (enhancement and inhibition) remain poorly characterized, making further progress difficult at this time.

## Supporting Information

Figure S1 ROC-type curves from rigid docking of metabolites/P-gp binders data set. Glide SP and XP with inner docking box coordinates (19.1, 52.3, −0.3) centered on the original ligand are in black and green, respectively. Glide SP and XP results with the inner box coordinates (19.0, 46.0, −6.0), located deeper in the cavity, are in red and blue, respectively.
(TIF)

Figure S2 Top-ranked poses from flexible docking. Both Glide XP and MM-GB/SA identified the similar poses as top ranked for digoxin (**A**) and loperamide (**B**) and the same pose for etoposide (**C**). (only Glide XP pose is shown for clarity). The different top-scored poses identified by the two methods for doxorubicin are shown in **D**. QZ59 is shown for reference in light green. For etoposide, two residues, Tyr303 and Ser725, forming hydrogen-bonding interactions with the ligand are shown. Residues Phe71, Phe953, and Phe974, positioned for cation-pi interaction, are shown for loperamide.
(TIF)

Figure S3 ROC-type curves (MM-GB/SA scoring) for metabolites/P-gp binders set (A) and Doan *et al.* dataset (B). Default treatment of protonation states (see Methods).
(TIF)

Figure S4 ROC-type curves (MM-GB/SA scoring) for flexible docking of Doan *et al.* dataset using the default treatment of protonation states (labeled as “pH = 7”) and when treating all compounds as neutral.
(TIF)

Figure S5 Flexible docking binding scores (MM-GB/SA) plotted versus P_active_ (**A**) and efflux ratio (**B**) for the Doan *et al.* dataset (pH 7).
(TIF)

Figure S6 Flexible docking binding scores (MM-GB/SA) plotted versus P_active_ (**A**) and efflux ratio (**B**) for the Doan *et al.* dataset (neutral).
(TIF)

Figure S7 Free energy of desolvation versus MM-GB/SA binding scores.
(TIF)

Table S1 Docking scores of P-gp binders selected from Hennessy *et al.*, 2007, Table 1.
(DOCX)

Table S2 Mouse P-gp binding cavity residues that were optimized with Prime in the first IFD round.
(DOCX)

Table S3 Docking scores of metabolites selected from KEGG database.
(DOCX)

Table S4 Docking scores for compounds from the Doan *et al.* dataset (at pH 7).
(DOCX)

Table S5 Docking scores for compounds (treated as neutral) from the Doan *et al.* dataset.
(DOCX)

Table S6 Binding cavity residues within 5 Å of QZ59 ligand in the original crystal structure and in the top flexible receptor docking pose.
(DOCX)

Text S1 Supplementary methods.
(RTF)
