# Supplementary figures and images for: Predicting Binding to P-Glycoprotein by Flexible Receptor Docking
Source: PLoS Comput Biol. 2011 Jun 23;7(6):e1002083. doi: 10.1371/journal.pcbi.1002083 (PMC3121697; doi:10.1371/journal.pcbi.1002083)

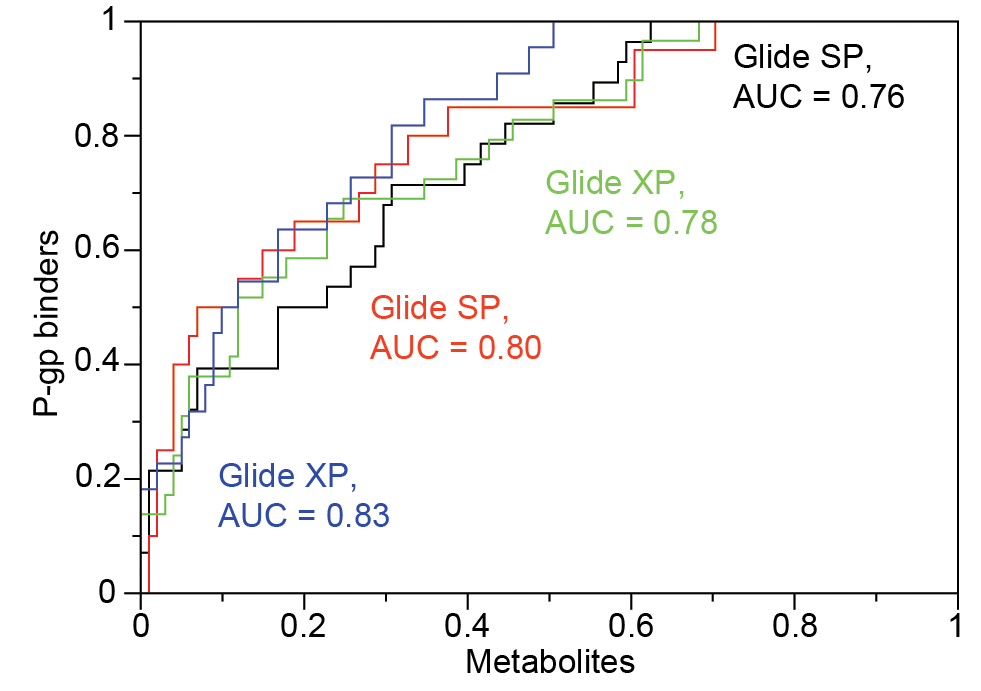

Supplement: Figure S1 — ROC-type curves from rigid docking of metabolites/P-gp binders data set. Glide SP and XP with inner docking box coordinates (19.1, 52.3, −0.3) centered on the original ligand are in black and green, respectively. Glide SP and XP results with the inner box coordinates (19.0, 46.0, −6.0), located deeper in the cavity, are in red and blue, respectively. (TIF) [file pcbi.1002083.s001.tif]

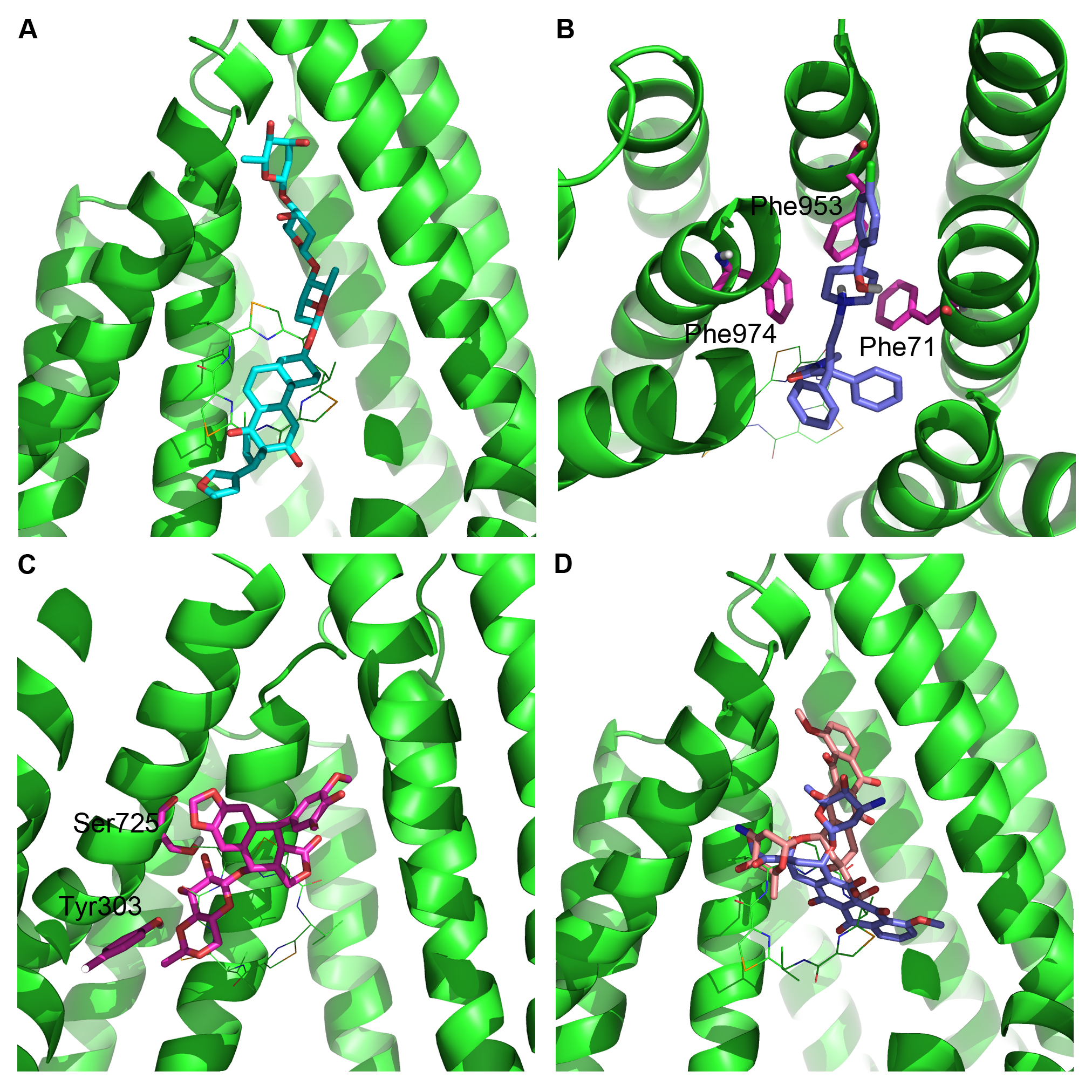

Supplement: Figure S2 — Top-ranked poses from flexible docking. Both Glide XP and MM-GB/SA identified the similar poses as top ranked for digoxin (A) and loperamide (B) and the same pose for etoposide (C). (only Glide XP pose is shown for clarity). The different top-scored poses identified by the two methods for doxorubicin are shown in D. QZ59 is shown for reference in light green. For etoposide, two residues, Tyr303 and Ser725, forming hydrogen-bonding interactions with the ligand are shown. Residues Phe71, Phe953, and Phe974, positioned for cation-pi interaction, are shown for loperamide. (TIF) [file pcbi.1002083.s002.tif]

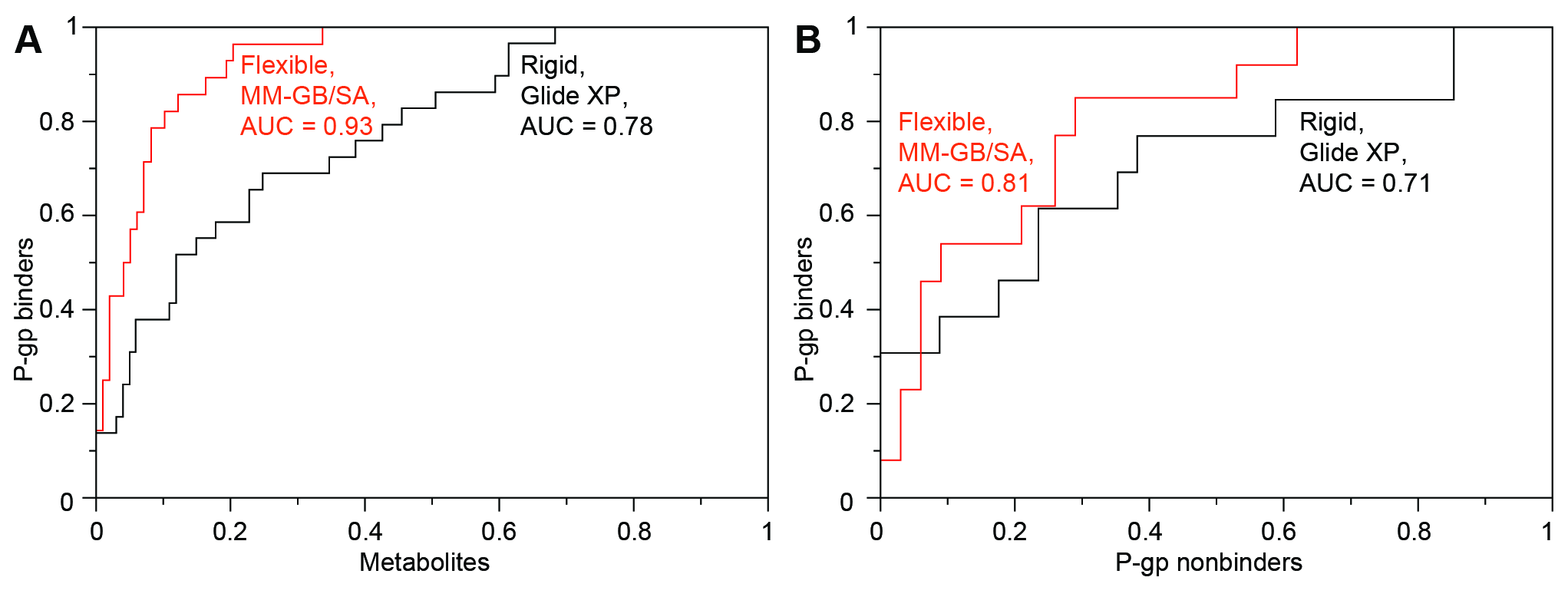

Supplement: Figure S3 — ROC-type curves (MM-GB/SA scoring) for metabolites/P-gp binders set (A) and Doan et al. dataset (B). Default treatment of protonation states (see Methods). (TIF) [file pcbi.1002083.s003.tif]

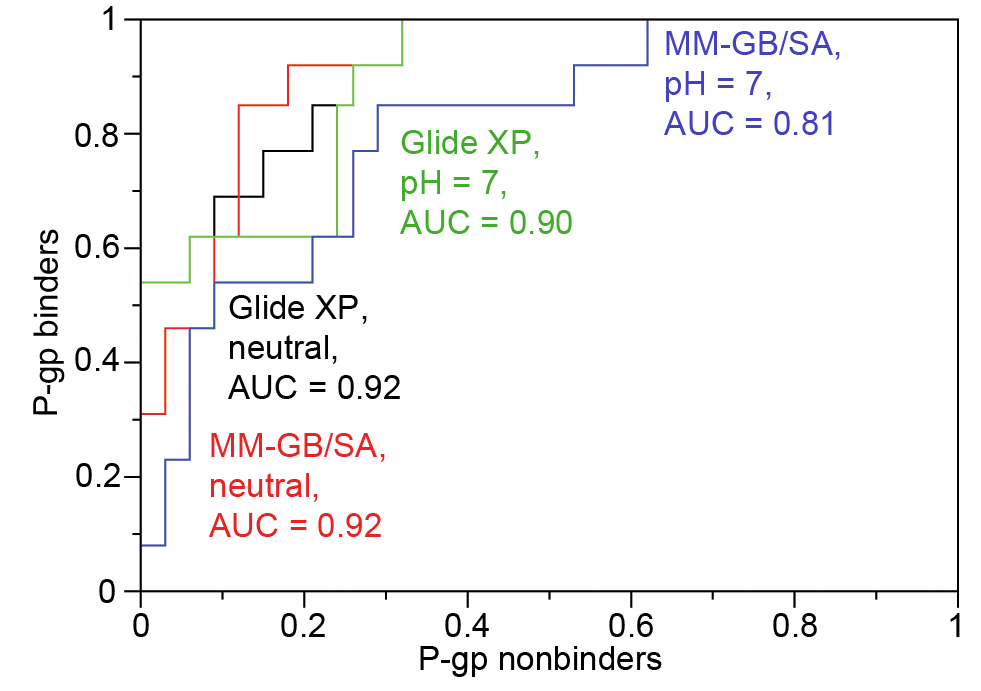

Supplement: Figure S4 — ROC-type curves (MM-GB/SA scoring) for flexible docking of Doan et al. dataset using the default treatment of protonation states (labeled as “pH = 7”) and when treating all compounds as neutral. (TIF) [file pcbi.1002083.s004.tif]

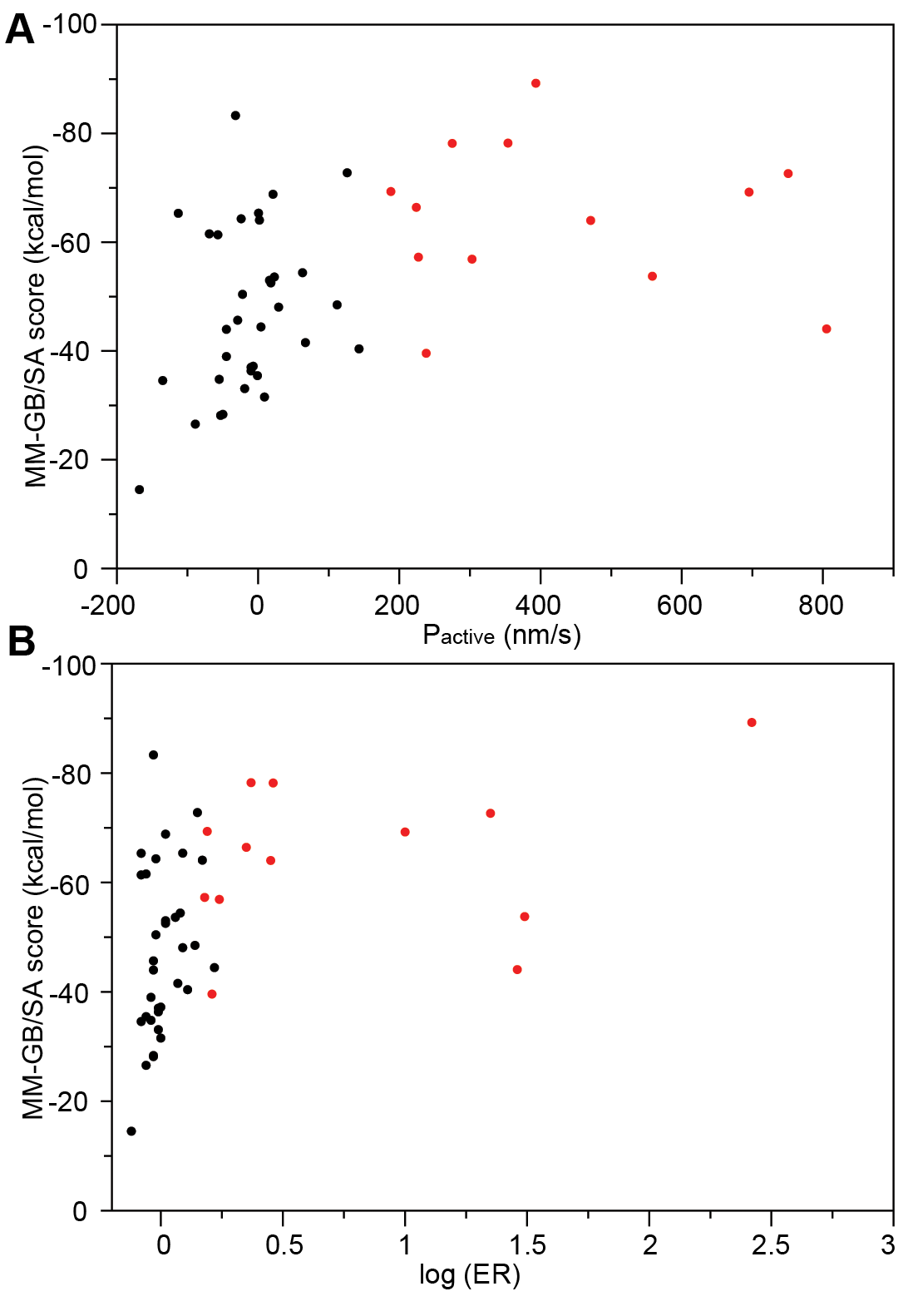

Supplement: Figure S5 — Flexible docking binding scores (MM-GB/SA) plotted versus Pactive (A) and efflux ratio (B) for the Doan et al. dataset (pH 7). (TIF) [file pcbi.1002083.s005.tif]

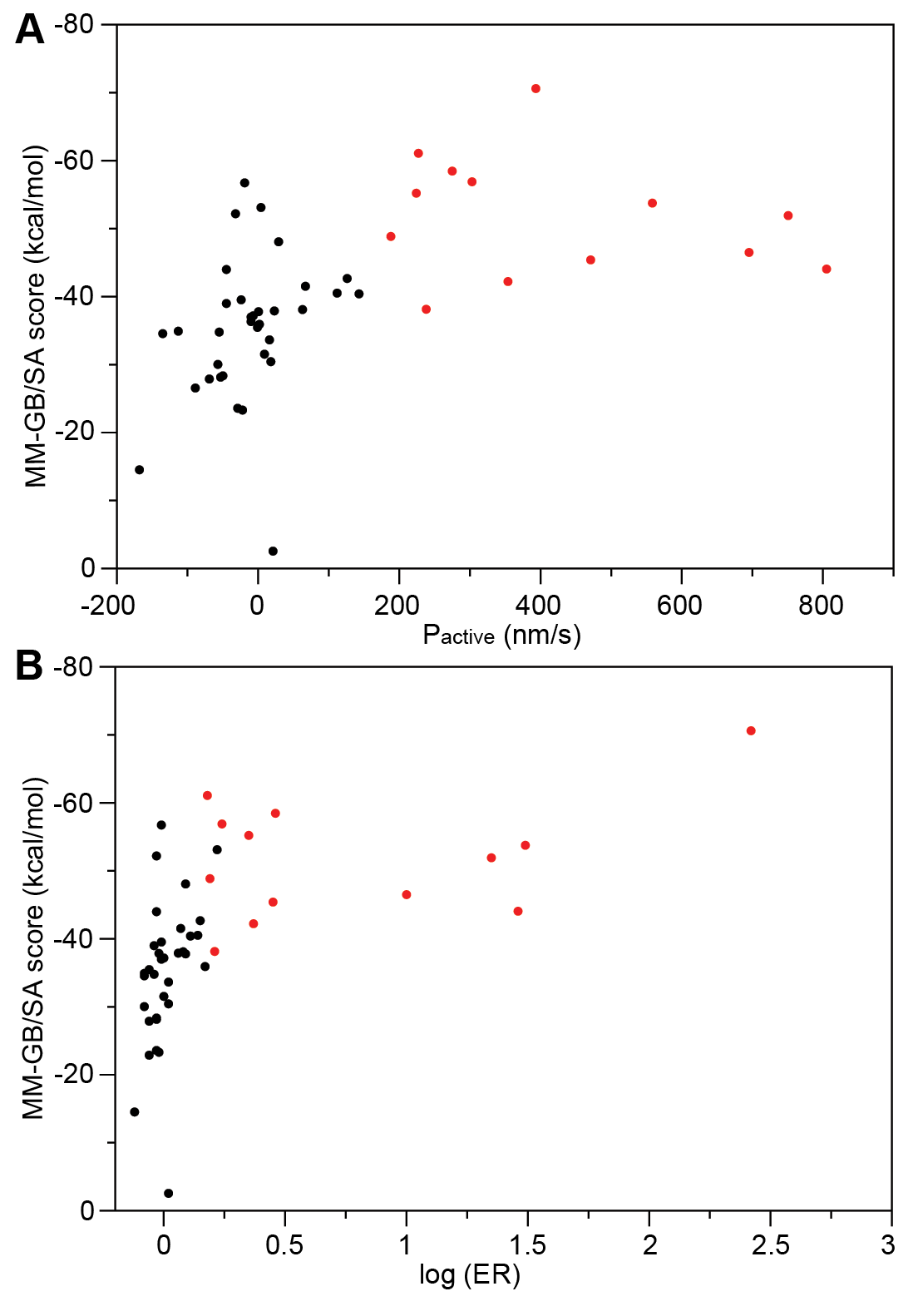

Supplement: Figure S6 — Flexible docking binding scores (MM-GB/SA) plotted versus Pactive (A) and efflux ratio (B) for the Doan et al. dataset (neutral). (TIF) [file pcbi.1002083.s006.tif]

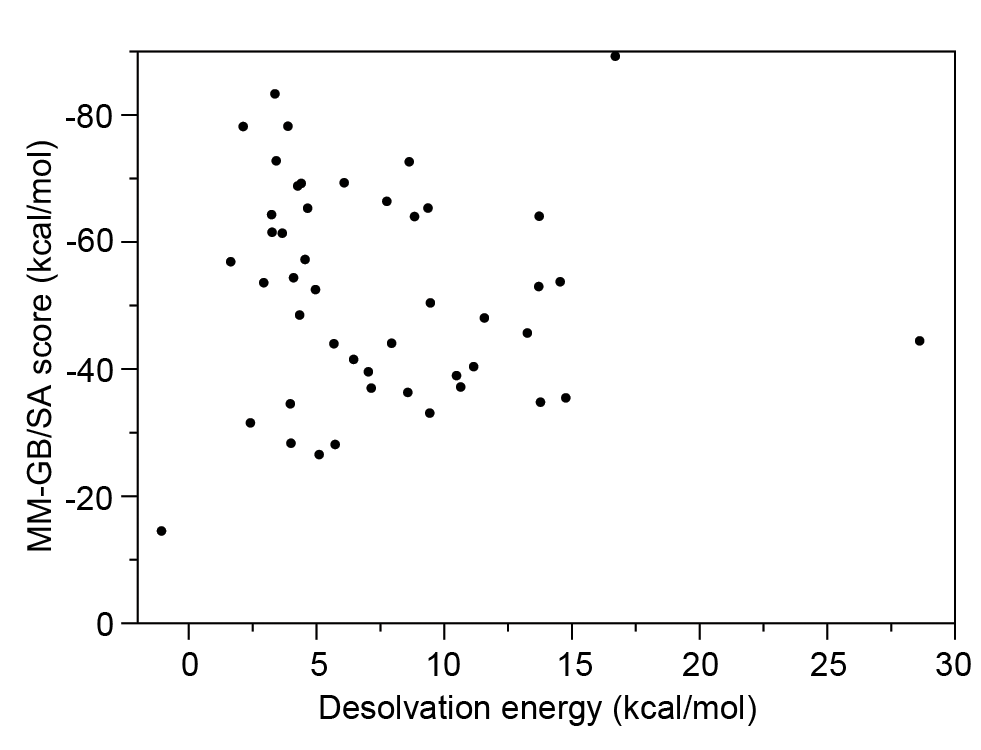

Supplement: Figure S7 — Free energy of desolvation versus MM-GB/SA binding scores. (TIF) [file pcbi.1002083.s007.tif]
