# Supplementary material for: Predicting Binding to P-Glycoprotein by Flexible Receptor Docking
Source: PLoS Comput Biol. 2011 Jun 23;7(6):e1002083. doi: 10.1371/journal.pcbi.1002083 (PMC3121697; doi:10.1371/journal.pcbi.1002083)
Supplement: Table S1 — Docking scores of P-gp binders selected from Hennessy et al., 2007, Table 1. (DOCX) [file pcbi.1002083.s008.docx]

| **P-gp Binders** | **Charge** | **Rigid**  **Glide XP (kcal/mol)** | **Flexible Glide XP (kcal/mol)** | **Flexible MM-GB/SA (kcal/mol)** | **MW* (g/mol)** |
| --- | --- | --- | --- | --- | --- |
| Aldosterone | 0 | -10.8 | -11.7 | -41.6 | 360.4 |
| Amprenavir | 0 | -11.1 | -13.7 | -44.1 | 505.6 |
| Colchicine | 0 | -9.3 | -11.4 | -50.1 | 399.4 |
| Corticosterone | 0 | -11.0 | -14.4 | -49.3 | 346.5 |
| Cortisol | 0 | -8.7 | -14.8 | -54.4 | 362.5 |
| Daunorubicin | 0 | -10.9 | -13.7 | -46.7 | 527.5 |
| Dexamethasone | 0 | -9.2 | -13.7 | -46.9 | 392.5 |
| Digoxin | 0 | -11.6 | -17.1 | -66.8 | 780.9 |
| Domperidone | 0 | -7.6 | -14.9 | -53.8 | 425.9 |
| Doxorubicin | 0 | -11.3 | -16.7 | -59.7 | 543.5 |
| Erythromycin | 0 | -8.2 | -12.2 | -42.7 | 733.9 |
| Etoposide | 0 | -5.5 | -17.5 | -58.5 | 588.6 |
| Hydrocortisone | 0 | -10.4 | -14.3 | -52.2 | 362.5 |
| Indinavir | 0 | -11.8 | -17.4 | -64.7 | 613.8 |
| Loperamide | 1 | -9.7 | -15.6 | -69.2 | 477.0 |
| Lovastatin | 0 | -11.9 | -13.9 | -48.9 | 404.5 |
| Nelfinavir | 1 | -8.9 | -15.6 | -72.6 | 567.8 |
| (R)-Ondansetron | 0 | -10.0 | -12.3 | -45.0 | 293.4 |
| (S)-Ondansetron | 0 | -9.2 | -12.3 | -43.4 | 293.4 |
| Quinidine | 1 | -7.00 | -12.3 | -58.0 | 324.4 |
| Ritonavir | 0 | -10.4 | -14.2 | -57.3 | 720.9 |
| Saquinavir | 1 | -10.0 | -17.8 | -89.3 | 670.8 |
| Teniposide | 0 | -8.8 | -15.6 | -60.8 | 656.6 |
| (R)-terfenadine | 1 | -12.5 | -17.2 | -78.2 | 471.7 |
| (S)-terfenadine | 1 | -10.7 | -15.9 | -68.5 | 471.7 |
| Vinblastine | 1 | -7.5 | -9.1 | -53.6 | 811.0 |
