# Supplementary material for: Predicting Binding to P-Glycoprotein by Flexible Receptor Docking
Source: PLoS Comput Biol. 2011 Jun 23;7(6):e1002083. doi: 10.1371/journal.pcbi.1002083 (PMC3121697; doi:10.1371/journal.pcbi.1002083)
Supplement: Table S2 — Mouse P-gp binding cavity residues that were optimized with Prime in the first IFD round. (DOCX) [file pcbi.1002083.s009.docx]

| Gly61 |
| --- |
| Leu64 |
| Met68 |
| Phe71 |
| Thr75 |
| Phe78 |
| Phe190 |
| Gln191 |
| Tyr303 |
| Tyr306 |
| Phe310 |
| Trp311 |
| Leu328 |
| Phe332 |
| Leu335 |
| Ile336 |
| Phe339 |
| Gln721 |
| Phe724 |
| Ser725 |
| Phe728 |
| Ser729 |
| Val731 |
| Val732 |
| Gly733 |
| Thr736 |
| Leu758 |
| Ile864 |
| Met945 |
| Tyr949 |
| Phe953 |
| Glu968 |
| Val970 |
| Leu971 |
| Phe974 |
| Ser975 |
| Ile977 |
| Val978 |
| Met982 |
