# Supplementary material for: Predicting Binding to P-Glycoprotein by Flexible Receptor Docking
Source: PLoS Comput Biol. 2011 Jun 23;7(6):e1002083. doi: 10.1371/journal.pcbi.1002083 (PMC3121697; doi:10.1371/journal.pcbi.1002083)
Supplement: Table S3 — Docking scores of metabolites selected from KEGG database. (DOCX) [file pcbi.1002083.s010.docx]

| **Metabolites** | **Charge** | **Rigid Glide XP (kcal/mol)** | **Flexible Glide XP (kcal/mol)** | **Flexible MM-GB/SA (kcal/mol)** |
| --- | --- | --- | --- | --- |
| Acetic acid | -1 | -3.8 | -4.8 | 2.6 |
| Acetylcholine | 1 | -5.3 | -6.8 | -51.1 |
| Adenine | 0 | -11.5 | -7.2 | -22.9 |
| Adenosine | 0 | -8.8 | -9.9 | -28.2 |
| Alanine | 0 | -3.6 | -4.1 | -27.8 |
| Ammonia | 1 | -2.9 | -4.2 | -9.6 |
| AMP | -1 | -8.7 | -10.6 | -16.0 |
| Arginine | 1 | -4.5 | -5.1 | -39.8 |
| Ascorbic acid | -1 | -7.6 | -7.9 | -6.6 |
| Asparagine | 0 | -4.4 | -5.2 | -23.2 |
| Aspartic acid | 0 | -5.3 | -5.2 | -6.6 |
| ATP | -3 | -7.5 | -8.8 | 15.0 |
| Bilirubin | -1 | -10.3 | -15.0 | -41.1 |
| Biotin | -1 | -7.3 | -8.8 | -30.6 |
| Butyric acid | -1 | -5.1 | -5.3 | -4.3 |
| Carnosine_a* | 0 | -5.0 | -9.6 | -38.1 |
| Carnosine_b* | 0 | -5.5 | -7.8 | -31.3 |
| Cholesterol** | 0 | -10.4 | -13.3 | -42.2 |
| Cholic acid | 0 | -10.8 | -13.9 | -62.0 |
| Citrate | -2 | -5.9 | -7.1 | 16.0 |
| Creatinine | 0 | -5.8 | -6.3 | -30.4 |
| CTP | -3 | -8.8 | -9.8 | 1.2 |
| Cysteine | 0 | -3.9 | -9.3 | -41.6 |
| Cytidine | 0 | -8.7 | -10.4 | -32.7 |
| Cytosine | 0 | -5.6 | -7.2 | -24.8 |
| D-galactosamine | 0 | -8.2 | -9.5 | -24.0 |
| D-Gluconic acid | -1 | -6.0 | -9.4 | -9.1 |
| D-Glucose | 0 | -8.5 | -10.2 | -31.4 |
| D-Mannose | 0 | -8.3 | -10.3 | -39.8 |
| dAMP | -1 | -8.6 | -10.1 | -23.4 |
| dATP | -3 | -9.2 | -8.9 | -0.9 |
| dCTP | -3 | -9.4 | -9.2 | 8.6 |
| Deoxyribose | 0 | -7.4 | -9.1 | -31.4 |
| Dopa | 0 | -8.5 | -9.8 | -44.4 |
| Dopamine | 1 | -8.7 | -9.5 | -52.8 |
| Epinephrine | 0 | -8.5 | -10.8 | -30.8 |
| FAD | -2 | -9.2 | -14.2 | -42.6 |
| Folic acid_a* | -2 | -8.2 | -14.0 | -12.4 |
| Folic acid_b* | -2 | -9.4 | -11.7 | -18.8 |
| Formic acid | -1 | -3.9 | -5.1 | -0.6 |
| Fructosamine | 0 | -8.0 | -10.0 | -33.9 |
| Fructose | 0 | -7.5 | -9.4 | -35.6 |
| Fumarate | -2 | -3.6 | -4.8 | 35.0 |
| Gamma-Aminobutyric acid (GABA) | 0 | -4.1 | -4.3 | -8.6 |
| Glutamic acid | -1 | -4.3 | -5.4 | -11.7 |
| Glutamine | 0 | -2.7 | -5.4 | -21.9 |
| Glutathione | -1 | -4.3 | -10.4 | -55.5 |
| Glyceraldehyde | 0 | -6.0 | -6.0 | -22.7 |
| Glycerol | 0 | -5.1 | -6.4 | -25.1 |
| Glycine | 0 | -3.0 | -7.5 | -35.2 |
| Guanine | 0 | -7.6 | -9.0 | -24.3 |
| Guanosine | 0 | -9.2 | -11.5 | -43.0 |
| Histamine_a* | 1 | -5.8 | -6.4 | -37.0 |
| Histamine_b* | 1 | -5.8 | -6.4 | -37.0 |
| Histidine_a* | 0 | -4.7 | -7.2 | -9.2 |
| Histidine_b* | 0 | -4.9 | -6.6 | -33.2 |
| Histidine_c* | 0 | -5.8 | -7.5 | -24.1 |
| Inosine | 0 | -9.2 | -12.3 | -38.5 |
| Isocitrate_a* | -2 | -6.1 | -7.1 | 16.2 |
| Isocitrate_b* | -2 | -6.0 | -7.1 | 25.7 |
| Isocitrate_c* | -2 | -6.8 | -7.0 | 19.1 |
| Isoleucine | 0 | -5.5 | -6.0 | -42.4 |
| Lactic acid | -1 | -5.3 | -6.6 | -7.4 |
| Lactose | 0 | -3.8 | -12.3 | -22.4 |
| Leucine | 0 | -5.3 | -6.1 | -40.3 |
| Leukotriene C4 | -2 | -11.4 | -16.8 | -43.7 |
| Lysine | 1 | -2.7 | -5.6 | -56.2 |
| Malic acid | -1 | -4.1 | -6.5 | 37.3 |
| Melanin | 0 | -9.6 | -12.2 | -37.9 |
| Melatonin | 0 | -9.2 | -10.1 | -40.0 |
| Methionine | 0 | -4.3 | -4.8 | -43.4 |
| Mevalonate | -1 | -6.0 | -6.5 | 1.2 |
| Muramic | 0 | -5.5 | -8.5 | -15.7 |
| Myristic acid | -1 | -7.0 | -8.2 | -19.7 |
| NAD | -1 | -10.0 | -14.5 | -44.1 |
| Neuraminic acid | 0 | -9.2 | -10.8 | -36.1 |
| Nicotinic acid | 0 | -4.1 | -7.1 | -13.2 |
| Oxaloacetate | -2 | -4.4 | -5.6 | 30.7 |
| Phenylalanine | 0 | -7.7 | -6.7 | -31.3 |
| Phosphoenolpyruvate (PEP) | -1 | -5.2 | -5.8 | 19.2 |
| Progesterone** | 0 | -9.2 | -13.0 | -37.7 |
| Proline | 0 | -4.9 | -5.9 | -22.5 |
| Prostaglandin F2A (dinoprost) | -1 | -11.0 | -14.0 | -35.6 |
| Pyruvic acid | -1 | -3.6 | -4.4 | 8.1 |
| Retinol | 0 | -10.3 | -13.1 | -40.8 |
| Riboflavin | 0 | -11.5 | -15.0 | -43.2 |
| Ribose | 0 | -7.9 | -7.7 | -17.4 |
| S-Adenosylmethioninamine (SAM) | 2 | -11.3 | -11.4 | -69.3 |
| Serine | 0 | -3.5 | -9.5 | -42.5 |
| Serotonin | 1 | -8.4 | -9.9 | -54.2 |
| Succinic acid | -1 | -5.1 | -6.1 | 31.5 |
| Sucrose | 0 | -10.2 | -12.6 | -38.3 |
| Taurine | 0 | -2.3 | -4.5 | -1.9 |
| Testosterone | 0 | -10.3 | -12.0 | -38.0 |
| Thiamin | 1 | -8.7 | -9.6 | -45.8 |
| Threonine | 0 | -3.3 | -6.9 | -45.3 |
| Thymidine | 0 | -6.9 | -9.3 | -42.1 |
| Thymine | 0 | -6.0 | -6.9 | -24.0 |
| Thyroxine** | 0 | -6.5 | -12.6 | -70.8 |
| Triacylglycerol | 0 | -4.7 | -6.1 | -36.2 |
| Tryptophan | 0 | -9.1 | -10.7 | -38.2 |
| Tyrosine | 0 | -7.5 | -8.0 | -47.8 |
| Uracil | 0 | -5.6 | -6.7 | -22.4 |
| Urea | 0 | -3.2 | -4.8 | -15.0 |
| Uridine | 0 | -6.9 | -9.0 | -24.0 |
| Valine | 0 | -5.4 | -6.3 | -22.8 |
| Vitamin D3** | 0 | -10.5 | -14.8 | -54.7 |
| Water | 0 | -4.4 | -4.0 | -7.6 |
| Xanthine | 0 | -6.0 | -7.8 | -26.2 |

** Highlighted in pink are metabolites reclassified as P-gp binders based on the reports in the literature.
