# Supplementary material for: Predicting Binding to P-Glycoprotein by Flexible Receptor Docking
Source: PLoS Comput Biol. 2011 Jun 23;7(6):e1002083. doi: 10.1371/journal.pcbi.1002083 (PMC3121697; doi:10.1371/journal.pcbi.1002083)
Supplement: Table S4 — Docking scores for compounds from the Doan et al. dataset (at pH 7). (DOCX) [file pcbi.1002083.s011.docx]

| **Name** | **P-gp interaction** | **Charge** | **Rigid**  **Glide XP**  **(kcal/mol)** | **Flexible Glide XP (kcal/mol)** | **Flexible MM-GB/SA (kcal/mol)** |
| --- | --- | --- | --- | --- | --- |
| Amprenavir | Y | 0 | -11.1 | -13.7 | -44.1 |
| Astemizole | Y | 1 | -11.7 | -15.2 | -66.4 |
| Diltiazem | Y | 1 | -7.8 | -12.2 | -57.3 |
| Domperidone | Y | 0 | -7.6 | -14.9 | -53.8 |
| (R)-Levomeprazine | Y | 1 | -6.0 | -11.8 | -69.3 |
| (S)-Levomeprazine | Y | 1 | -10.0 | -12.0 | -61.4 |
| Loperamide | Y | 1 | -9.7 | -15.6 | -69.2 |
| (R)-Mequitazine | Y | 1 | -10.3 | -12.1 | -64.0 |
| (S)-Mequitazine | Y | 1 | -9.6 | -12.5 | -61.1 |
| Nelfinivir | Y | 1 | -8.9 | -15.6 | -72.6 |
| Protriptylene | Y | 1 | -10.4 | -12.7 | -78.2 |
| Risperidone | Y | 1 | -9.8 | -12.7 | -39.6 |
| Saquinavir | Y | 1 | -10.0 | -17.8 | -89.3 |
| (R)-Terfenadine | Y | 1 | -12.5 | -17.2 | -78.2 |
| (S)-Terfenadine | Y | 1 | -10.7 | -15.9 | -68.5 |
| (R)-Verapamil | Y | 0 | -11.4 | -14.7 | -56.9 |
| (S)-Verapamil | Y | 0 | -11.2 | -13.3 | -45.5 |
| Amantadine | N | 1 | -8.2 | -9.2 | -50.4 |
| Antipyrine | N | 0 | -6.4 | -9.2 | -28.3 |
| (R)-Atenolol | N | 1 | -8.4 | -9.9 | -64.4 |
| (S)-Atenolol | N | 1 | -8.8 | -9.9 | -65.4 |
| (R)-Biperiden | N | 1 | -8.5 | -12.7 | -60.6 |
| (S)-Biperiden | N | 1 | -8.0 | -13.3 | -64.3 |
| Carbamazepine | N | 0 | -9.4 | -11.3 | -36.3 |
| (R)-Chlorpheniramine | N | 1 | -9.9 | -10.8 | -83.3 |
| (S)-Chlorpheniramine | N | 1 | -9.5 | -11.1 | -60.0 |
| Clonidine | N | 0 | -8.3 | -9.4 | -37.2 |
| (R)-Doxylamine | N | 1 | -9.4 | -11.1 | -54.0 |
| (S)-Doxylamine | N | 1 | -9.7 | -11.6 | -61.4 |
| Flumazenil | N | 0 | -8.9 | -10.5 | -39.0 |
| Guanabenz | N | 0 | -10.3 | -9.8 | -34.8 |
| Guanfacine | N | 0 | -9.8 | -11.6 | -48.1 |
| Imipramine | N | 1 | -9.2 | -12.1 | -68.8 |
| Indomethacin | N | -1 | -9.8 | -13.7 | -33.1 |
| (R)-Ketamine | N | 0 | -9.2 | -10.0 | -25.9 |
| (S)-Ketamine | N | 0 | -9.8 | -10.2 | -28.2 |
| Lidocaine | N | 1 | -5.0 | -10.0 | -65.3 |
| Mannitol | N | 0 | -8.5 | -10.0 | -35.5 |
| Mephentermine | N | 1 | -8.3 | -9.5 | -61.6 |
| Meprobamate | N | 0 | -7.3 | -8.1 | -37.0 |
| (R)-Metoprolol | N | 1 | -9.5 | -9.6 | -45.8 |
| (S)-Metoprolol | N | 1 | -10.2 | -10.1 | -54.4 |
| (R)-Mexilitine | N | 0 | -8.3 | -9.5 | -22.9 |
| (S)-Mexilitine | N | 0 | -8.2 | -8.9 | -26.6 |
| Naloxone | N | 0 | -9.0 | -13.2 | -40.4 |
| Naltrexone | N | 1 | -6.1 | -13.0 | -53.0 |
| Nitrazepam | N | 0 | -9.9 | -11.3 | -41.5 |
| Nordazepam | N | 0 | -10.2 | -13.7 | -44.0 |
| (R)-Oxprenolol | N | 1 | -8.2 | -9.6 | -45.0 |
| (S)-Oxprenolol | N | 1 | -8.3 | -9.3 | -48.5 |
| (R)-Pheniramine | N | 1 | -9.4 | -11.4 | -69.5 |
| (S)-Pheniramine | N | 1 | -8.0 | -11.3 | -72.8 |
| (R)-Propranolol | N | 1 | -8.5 | -11.5 | -39.3 |
| (S)-Propranolol | N | 1 | -10.3 | -13.0 | -52.5 |
| Scopolamine | N | 1 | -7.4 | -12.1 | -53.6 |
| (R)-Selegiline | N | 0 | -9.2 | -9.1 | -14.5 |
| (S)-Selegiline | N | 0 | -9.2 | -7.6 | -13.7 |
| Sulfasalazine_a* | N | -1 | -10.3 | -14.4 | -44.4 |
| Sulfasalazine_b* | N | -1 | -9.7 | -11.7 | -27.7 |
| Sumatriptan | N | 1 | -10.8 | -12.5 | -64.1 |
| Tacrine | N | 1 | -9.4 | -10.1 | -45.7 |
| (R)-Warfarin | N | 0 | -10.5 | -12.3 | -34.6 |
| (S)-Warfarin | N | 0 | -10.5 | -12.9 | -33.8 |
| Zimeldine | N | 0 | -10.5 | -11.0 | -31.5 |
