# Supplementary material for: Predicting Binding to P-Glycoprotein by Flexible Receptor Docking
Source: PLoS Comput Biol. 2011 Jun 23;7(6):e1002083. doi: 10.1371/journal.pcbi.1002083 (PMC3121697; doi:10.1371/journal.pcbi.1002083)
Supplement: Table S6 — Binding cavity residues within 5 Å of QZ59 ligand in the original crystal structure and in the top flexible receptor docking pose. (DOCX) [file pcbi.1002083.s013.docx]

| Residues interacting w/ QZ59-RRR (Aller et al., Table S3) | Within 5 Å of the IFD pose |
| --- | --- |
| Met68 | x |
| Tyr303 | x |
| Phe332 | x |
| Leu335 | x |
| Ile336 | x |
| Phe339 |  |
| Gln721 | x |
| Phe724 | x |
| Phe728 | x |
| Tyr949 |  |
| Phe974 | x |
| Ser975 | x |
| Val978 | x |
