# Supplementary material for: Predicting Binding to P-Glycoprotein by Flexible Receptor Docking
Source: PLoS Comput Biol. 2011 Jun 23;7(6):e1002083. doi: 10.1371/journal.pcbi.1002083 (PMC3121697; doi:10.1371/journal.pcbi.1002083)
Supplement: Text S1 — Supplementary methods. (RTF) [file pcbi.1002083.s014.rtf]

Supplementary Methods

Chemical shifts are reported in  units (ppm) relative to TMS as an internal standard. Coupling constants (J) are reported in Hertz (Hz). Characterization data are reported as follows: chemical shift, multiplicity (s=singlet, d=doublet, t=triplet, q=quartet, br=broad, m=multiplet), coupling constants, number of protons, mass to charge ratio.

Benzyl N-[(1S)-1-[(cyanomethyl)carbamoyl]-2-(dimethylamino)ethyl]carbamate (2) was prepared in 47% yield over two steps according to the general procedure described in Synthetic Methods and employing N,N-dimethyltrimethylsilylamine (2 equivalents) in the first reaction. 

1H NMR (400 MHz, CDCl3)  9.10 (br. s., 1H), 7.25 - 7.47 (m, 5H), 5.75 - 5.97 (m, 1H), 5.01 - 5.19 (m, 2H), 4.03 - 4.29 (m, 3H), 2.65 (d, J = 9.34 Hz, 1H), 2.44 - 2.56 (m, 1H), 2.34 (br. s., 6H);  13C NMR (100 MHz, CDCl3)  171.5, 156.0, 136.1, 128.6, 128.2, 128.1, 116.0, 67.1, 60.5, 50.7, 45.0, 27.2;   MS (m/z): [M+H]+ = 306 

Benzyl N-[(1S)-1-[(cyanomethyl)carbamoyl]-3-phenylpropyl]carbamate (3) was prepared in 29% according to the general procedure described in Synthetic Methods.

1H NMR (400 MHz, CDCl3)  7.29 - 7.40 (m, 4H), 7.22 - 7.29 (m, 2H), 7.15 - 7.22 (m, 2H), 7.09 - 7.15 (m, 2H), 5.60 (d, J = 8.24 Hz, 1H), 5.02 - 5.16 (m, 2H), 4.15 (m, 1H), 4.02 (d, J = 5.68 Hz, 2H), 2.58 - 2.74 (m, 2H), 2.08 - 2.21 (m, 1H), 1.96-1.91 (m, 1H);  13C NMR (100 MHz, CHLOROFORM-d)  172.0, 156.5, 140.1, 135.8, 128.6, 128.6, 128.4, 128.4, 128.0, 126.4, 115.8, 67.5, 54.2, 33.3, 31.6, 27.4;  MS (m/z): [M+H]+ = 352

Benzyl N-[(1S)-1-[(cyanomethyl)carbamoyl]-2-[methyl(phenyl)amino]ethyl] carbamate (4) was prepared in 18% yield over two steps according to the general procedure described in Synthetic Methods and employing N-methylaniline (10 equivalents) in the first reaction. 

1H NMR (400 MHz, CDCl3)  7.23 - 7.41 (m, 6H), 6.78 - 6.91 (m, 4H), 5.57 - 5.66 (m, 1H), 5.07 - 5.15 (m, 2H), 4.47 (s, 1H), 4.07 - 4.16 (m, 1H), 3.98 - 4.07 (m, 1H), 3.68 - 3.77 (m, 1H), 3.44 - 3.51 (m, 1H), 2.95 (s, 3H);  13C NMR (100 MHz, CHLOROFORM-d)  171.0, 156.0, 148.8, 135.8, 129.6, 129.6 128.6, 128.6 128.4, 128.1, 128.1, 118.5, 115.3, 113.3, 77.3, 77.2, 76.7, 67.4, 55.5, 52.5, 39.7, 27.5; MS (m/z): [M+H]+ = 367

Benzyl N-[(1S)-1-[(cyanomethyl)carbamoyl]-2-(1,2,3,4-tetrahydroquinolin-1-yl)ethyl]carbamate (5)  was prepared in 30% yield over two steps according to the general procedure described in Synthetic Methods and employing tetrahydroquinoline (3 equivalents) in the first reaction. 

1H NMR (400 MHz, CDCl3)  7.28 - 7.44 (m, 5H), 7.11 (br. s., 1H), 6.99 (d, J = 7.33 Hz, 1H), 6.90 (br. s., 1H), 6.84 (br. s., 1H), 6.69 (t, J = 7.33 Hz, 1H), 5.58 - 5.74 (m, 1H), 5.12 (s, 2H), 4.41 - 4.59 (m, 1H), 4.14 (dd, J = 6.04, 17.40 Hz, 1H), 4.00 (dd, J = 5.59, 17.49 Hz, 1H), 3.68 (d, J = 10.26 Hz, 1H), 3.40 (dd, J = 9.34, 14.47 Hz, 1H), 3.19 - 3.34 (m, 2H), 2.77 (t, J = 6.04 Hz, 2H), 1.88 - 1.99 (m, 2H);  13C NMR (100 MHz, CDCl3)  171.6, 156.4, 145.0, 136.1, 129.8, 128.8, 128.6, 128.2, 127.6, 123.4, 117.6, 115.8, 111.5, 67.5, 54.7, 52.6, 51.1, 28.0, 27.7, 22.4;  MS (m/z): [M+H]+ = 393

Benzyl N-[(1S)-1-[(cyanomethyl)carbamoyl]-2-(1H-indol-3-yl)ethyl]carbamate (6) was prepared in 60% yield according to the general procedure described in Synthetic Methods. 

1H NMR (400 MHz, DMSO-d6)  10.83 (br. s., 1H), 8.79 (t, J = 5.59 Hz, 1H), 7.62 (d, J = 7.87 Hz, 1H), 7.54 (d, J = 8.42 Hz, 1H), 7.23 - 7.36 (m, 6H), 7.14 (s, 1H), 7.04 - 7.09 (m, 1H), 6.95 - 7.00 (m, 1H), 4.95 (s, 2H), 4.27 (dd, J = 4.94, 9.71 Hz, 1H), 4.12 - 4.16 (m, 2H), 3.11 (dd, J = 4.94, 14.47 Hz, 1H), 2.93 (dd, J = 9.89, 14.47 Hz, 1H); 13C NMR (100 MHz, DMSO-d6)  172.6, 155.9, 136.9, 136.1, 128.3, 127.7, 127.5, 127.1, 123.9, 120.9, 118.4, 118.3, 117.6, 111.3, 109.8, 65.4, 55.3, 27.5, 27.2; MS (m/z): [M+H]+ = 377

Benzyl N-[(1S)-1-[(cyanomethyl)carbamoyl]-2-(2H-indazol-2-yl)ethyl]carbamate (7) was prepared in 19% yield over two steps according to the general procedure described in Synthetic Methods and employing indazole (1.05 equivalents) with heating at 60 oC in the first reaction. 

1H NMR (400 MHz, DMSO-d6)  8.95 (t, J = 5.31 Hz, 1H), 8.27 (s, 1H), 7.82 (d, J = 8.61 Hz, 1H), 7.69 (d, J = 8.61 Hz, 1H), 7.59 (d, J = 8.79 Hz, 1H), 7.15 - 7.32 (m, 5H), 7.01 - 7.06 (m, 1H), 4.89 - 4.99 (m, 2H), 4.80 (dd, J = 4.03, 13.00 Hz, 1H), 4.65 - 4.73 (m, 1H), 4.55 - 4.64 (m, 1H), 4.15 (d, J = 5.68 Hz, 2H); 13C NMR (100 MHz, CDCl3)  169.6, 156.3, 148.9, 135.6, 128.6, 128.5, 128.2, 127.2, 125.5, 122.3, 121.6, 120.6, 116.7, 115.2, 67.7, 55.3, 53.5, 27.5; MS: (m/z): [M+H]+ = 379

Benzyl N-[(1S)-1-[(cyanomethyl)carbamoyl]-2-(1H-indol-1-yl)ethyl]carbamate (8) was prepared in 4% yield over three steps according to the general procedure described in Synthetic Methods and employing indoline (1.05 equivalents) in the first reaction. 

1H NMR (400 MHz, CDCl3CHLOROFORM)  7.63 (d, J = 7.87 Hz, 1H), 7.30 - 7.46 (m, 5H), 7.22 (br. s., 1H), 7.10 - 7.18 (m, 1H), 6.97 - 7.05 (m, 1H), 6.53 (d, J = 3.11 Hz, 1H), 6.01 (br. s., 1H), 5.49 (br. s., 1H), 5.10 (s, 2H), 4.62 (br. s., 2H), 4.28 - 4.41 (m, 1H), 3.91 (dd, J = 5.77, 17.49 Hz, 1H), 3.77 (dd, J = 5.68, 17.40 Hz, 1H); 13C NMR (100 MHz, CDCl3)  169.9, 155.9, 135.9, 135.6, 128.7, 128.5, 128.1, 122.4, 121.3, 120.2, 115.0, 109.2, 102.8, 77.3, 76.7, 67.6, 54.5, 47.4, 27.4
MS: (m/z): [M+H]+ = 377


Benzyl N-[(1S)-1-[(cyanomethyl)carbamoyl]-2-(1H-imidazol-5-yl)ethyl]carbamate (9) was obtained in 20% yield according to the general procedure provided described in Synthetic Methods.

1H NMR (400 MHz, DMSO-d6)  11.80 (br. s., 1H), 8.65 (t, J = 5.40 Hz, 1H), 7.53 (s, 1H), 7.19 - 7.39 (m, 5H), 6.77 (br. s., 1H), 4.91 - 5.08 (m, 2H), 4.16 - 4.31 (m, 1H), 4.01 - 4.15 (m, 2H), 2.85-2.97 (m, 1H), 2.73-2.81 (m,1H); 13C NMR (100 MHz, DMSO-d6)  171.7, 155.8, 136.8, 134.5, 132.3, 128.4, 127.8, 127.6, 117.5, 116.6, 65.6, 54.4, 28.5, 27.2;  MS: (m/z): [M+H]+ = 328

Benzyl N-[(1S)-1-[(cyanomethyl)carbamoyl]-2-(1H-pyrazol-1-yl)ethyl]carbamate (10) was prepared in 13% yield over two steps according to the general procedure described in Synthetic Methods and employing pyrazole (1.05 equivalents) with heating at 55 oC in the first reaction. 

1H NMR (400 MHz, CDCl3)  7.53 (d, J = 1.83 Hz, 1H), 7.29 - 7.48 (m, 6H), 6.58 (d, J = 6.78 Hz, 1H), 6.28 (s, 1H), 5.15 (s, 2H), 4.58 - 4.76 (m, 2H), 4.41 (dd, J = 4.49, 13.64 Hz, 1H), 4.11 (dd, J = 5.95, 17.67 Hz, 1H), 4.00 (dd, J = 5.49, 17.40 Hz, 1H);  13C NMR (100 MHz, CDCl3)  170.0, 156.5, 140.7, 135.9, 131.6, 128.9, 128.7, 128.5, 115.5, 106.4, 67.8, 55.6, 52.3, 27.7; MS (m/z): [M+H]+ = 329
